# IL-7: a potential next-generation adjuvant for immune cell therapies

**DOI:** 10.3389/fimmu.2025.1736931

**Published:** 2026-01-02

**Authors:** Richard S. Hotchkiss, John F. DiPersio, Cassian Yee, Russell K. Pachynski, Marcel R. M. Van Den Brink

**Affiliations:** 1 Department of Anesthesiology, Medicine, and Surgery, Washington University School of Medicine, St. Louis, MO, United States; 2 Department of Medicine, Washington University School of Medicine, St Louis, MO, United States; 3 Department of Melanoma Medical Oncology, The University of Texas MD Anderson Cancer Center, Houston, TX, United States; 4 Department of Immunology, The University of Texas MD Anderson Cancer Center, Houston, TX, United States; 5 City of Hope Los Angeles and National Medical Center, Duarte, CA, United States

**Keywords:** cancer therapy, car-t, chimeric antigen receptor T-cell immunotherapy, IL-7, immunotherapy, TIL (tumor infiltrating lymphocytes)

## Abstract

Cell-based immune therapies ranging from CAR-T cells to tumor infiltrating lymphocytes (TILs) and endogenous T-cell products, have produced unprecedented clinical responses in hematologic malignancies and are currently under active investigation for solid tumors. Nevertheless, several key challenges continue to limit the durability and breadth of clinical benefit. IL-7 is a pleiotropic cytokine that increases both the number and function of lymphocytes. Although not yet clinically approved, IL-7 has been used in over 620 adult and pediatric patients for a variety of reasons including, for example, to hasten bone marrow recovery after allogenic stem cell transplantation, to reverse lymphopenia due to HIV and idiopathic etiologies, to treat patients with various malignancies, and to boost vaccine responses. IL-7 is generally well-tolerated and effective in producing a *durable* increase in the number and function of CD4 and CD8 T cells. Recently, IL-7 has been used clinically in multiple myeloma patients receiving CAR-T cell therapy, in patients with urothelial cancer who are receiving checkpoint inhibitors, in patients undergoing endogenous lymphocyte cell therapy, and in critically-ill lymphopenic patients with COVID-19. The authors, all of whom have used IL-7 clinically, discuss how IL-7 effectively addresses all the major problems currently limiting adoptive cell therapies. Peering into the future, we believe that IL-7 will be a major advance as an adjuvant treatment in many cell therapies and hope that this commentary will expedite IL-7’s testing in multiple clinical settings.

## Introduction

Adoptive cell therapies, including chimeric antigen receptor (CAR)-T cells, tumor-infiltrating lymphocytes (TILs), and endogenous T cell therapies, have transformed the treatment of hematologic malignancies and are increasingly being explored for solid tumors (1–4). Despite remarkable clinical responses, several problems persist which, if resolved, could lead to more durable remissions and cures. These include poor T cell expansion and persistence, development of novel tumor antigens, T cell exhaustion, impaired trafficking to tumor sites, and increased infectious complications (1–4). IL-2 has been used in an attempt to enhance the durability and breadth of the anti-tumor response but, its short half-life, expansion of T regulatory cells (Tregs), and toxicity have limited its utility (5).

**Figure 1:**
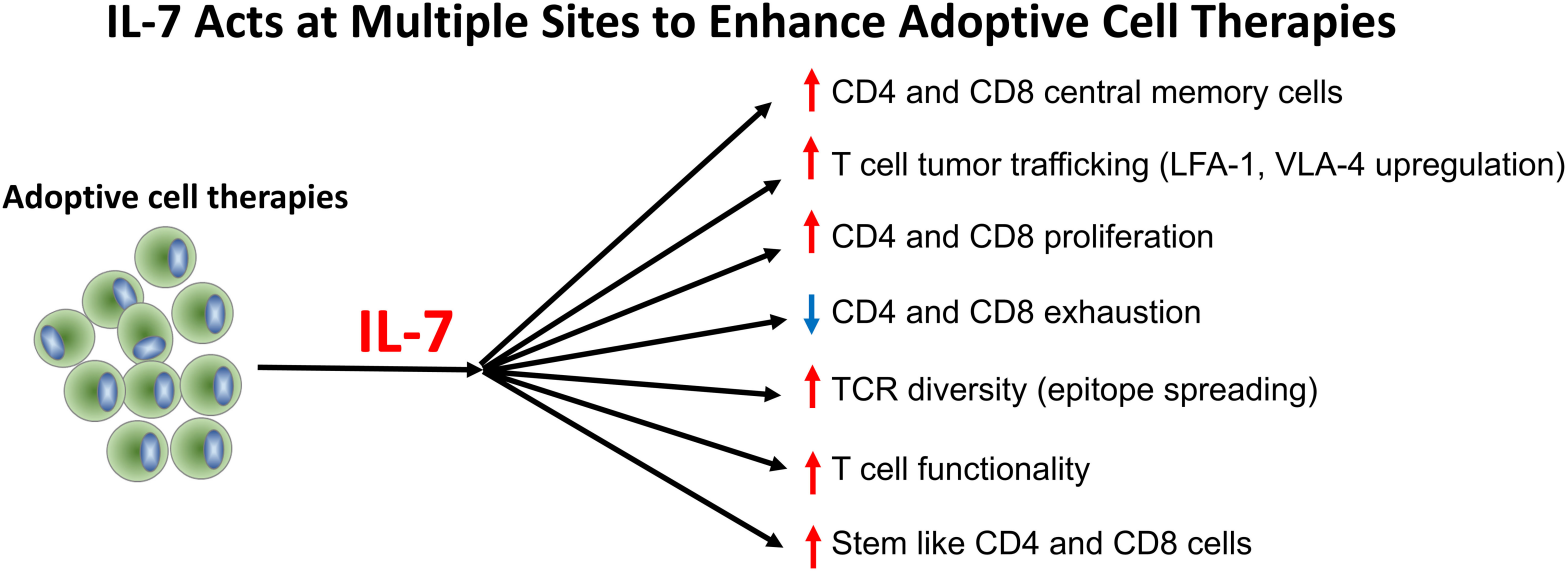
IL-7 acts at multiple sites to enhance CAR-T, TIL, and endogenous T cell therapies.

IL-7, like IL-2, is a member of the common-γ-chain cytokine family (5–8). Although not yet currently FDA approved, IL-7 has been used in over 620 adult and pediatric patients for a variety of reasons including, for example, to hasten bone marrow recovery after allogenic stem cell transplantation, to reverse lymphopenia due to HIV and idiopathic etiologies, to treat patients with various malignancies, to act as a vaccine adjuvant in dendritic cell vaccines in pediatric sarcoma, and in prostate cancer, to boost efficacy of checkpoint inhibitors in patients with urothelial cancer, and to treat various infectious disorders including sepsis and SARS-CoV-2 (9–17). IL-7 is generally well-tolerated and effective in producing a durable increase in the number and function of CD4 and CD8 T cells (7, 11, 18, 19). The authors, all of whom have used IL-7 clinically for different indications, believe that IL-7 offers great promise as a versatile, next-generation immune adjuvant and will decrease morbidity and improve survival in patients receiving various types of cell therapies. In this *Perspectives* article, the authors will describe how, in their opinion, and supported by evidence, IL-7 overcomes many of the key roadblocks to cell therapies as expertly presented in a previous *Blood Spotlight* article (1). We will also discuss how IL-7 offers many advantages over IL-2 and the potential adverse effects and contraindications to use of IL-7 (**Figure 1**, **Table 1**). Peering into the future, we believe that IL-7 will be a major advance as an adjuvant treatment in many cell therapies and hope that this commentary will expedite IL-7’s testing in multiple clinical settings.

**Table 1:** Comparison of IL-2, IL-7, and IL-15 effects on cell therapies.

Effects on:	IL-2	IL-7	IL-15
Cytokine Release Syndrome (CRS)	High dose IL-2 therapy causes CRS in majority of patients.	CRS unlikely because activated effector T cells downregulate IL-7Rα (CD127).	CRS possible/likely; IL-15Rβ is highly expressed on activated CD8 T and NK cells.
CD4 and CD8 T Cell Counts	Unpredictable effects on CD4 and CD8 T cell counts, dependent upon dose of IL-2.	Dose-dependent effect to produce durable increases in CD4 and CD8 T cells.	Increases CD8 T cells robustly but does not affect CD4 T cells.
T Cell Differentiation	IL-2 accelerates terminal differentiation towards effector cells.	IL-7 increases and sustains absolute numbers of naïve and memory CD4 and CD8 T cells.	Promotes differentiation toward effector memory and terminal effector CD8 T cells.
T Cell Receptor Diversity	IL-2 does not broaden TCR diversity.	IL-7 enhances TCR diversity and epitope spreading.	Expands existing effector clones but does not increase T cell receptor diversity.
Stem-Like T Cells	IL-2 expands stem-like T cells, particularly CD8+ T cells but only at low doses.	IL-7 expands stem-like CD4 and CD8 T cells and polyfunctionality.	Expands CD8+ T stem-like memory T cells.
Effector T Cells	Generates a robust number of effector cells but there is the risk of activation induced cell death.	Generates a robust number of CD4 and CD8 effector cells.	Expands effector CD8 T cells and NK cells.
T Cell Exhaustion	No effect on the reversal of exhaustion.	Reverses T cell exhaustion and restores polyfunctionality.	Limited ability to reverse exhaustion.
T Regulatory Cells	IL-2 drives expansion of Treg cells.	No effect on T reg cells.	Minimal effect on T reg cells.
Infections	High-dose IL-2 is associated with increased infection risk in cancer patients.	Data suggest that IL-7 helps to clear existing infections and reduces risk of acquiring new infections.	Less data available but existing data suggest that L-15 may reduce infections by activating CD8 T cells and NK cells.

## Advances in IL-7 design and production

One of the first clinical trials of IL-7 was conducted at the National Cancer Institute by Rosenberg and colleagues in which twelve patients with a variety of malignancies were treated with a non-glycosylated recombinant human IL-7 produced by *Escherichia coli* containing DNA encoding the human protein (CYT 99-007, Cytheris) (20). The IL-7 was “well tolerated in all patients” but significant levels of anti-IL-7 antibodies to CYT 99–007 were detected. Subsequently, Cytheris developed a new advanced version of IL-7, i.e., a fully glycosylated human recombinant IL-7 produced in a CHO cell line (CYT107) now developed by RevImmune. CYT107 was well tolerated in multiple clinical trials of over 600 patients. It has a circulating half-life of approximately 12 hours and has a very low incidence of inducing anti-drug antibodies (9–17). A second company, NeoImmuneTech, has produced a novel IL-7 preparation (efineptakin alfa, NT-I_7_) using a fusion protein to bind two human IL-7 proteins. NT-I_7_ has a very long half-life of approximately 63 hours (21). NT-I_7_ has been well-tolerated in multiple clinical trials but there is higher incidence of anti-drug antibodies compared to CYT107. Currently, there are no reports of any adverse effects from these anti-drug antibodies.

## IL-7 treatment induces a sustained increase in CD4 and CD8 T cells

IL-7 causes a dose-dependent increase in the absolute numbers of CD4 and CD8 T cells (5, 10–12). Unlike IL-2, which drives effector differentiation and may precipitate activation-induced death of CD4 and CD8 T cells, IL-7 preferentially expands naïve, central memory, and stem-like progenitor CD4 and CD8 T cells (5, 7, 18, 19). In addition to these peripheral effects, IL-7 is a non-redundant trophic factor for thymopoiesis, increasing thymic cellularity and recent-thymic-emigrant output (22–24). IL-7 also amplifies antigen-driven T cell responses and induces the transition of effector T cells to memory T cells (5, 7, 8, 18). The effect of IL-7 to increase lymphocyte counts is dose-dependent and many clinical trials demonstrate a robust 2–4 fold increase in both CD4 and CD8 T cells which frequently persists for weeks to months depending upon the clinical condition (5, 18). This effect of IL-7 to produce a sustained increase in circulating lymphocytes occurs across multiple lymphocyte subsets thereby ensuring a diverse and persistent T cell repertoire (9, 11, 15, 18, 19). The effect of IL-7 to cause a durable increase in lymphocytes is especially relevant for CAR-T cells in which studies show that the half-life of the circulating CAR-T cells directly correlates with improved outcomes (25, 26). In this regard, studies in a mouse myeloma model demonstrated that IL-7 markedly prolonged the persistence of the CAR-T cells and resulted in 100% long-term tumor-free survival (26). This effect almost certainly occurs for other adoptive lymphocyte therapies including TILs and engineered T cell receptor therapies and is due to IL-7’s effect to increase anti-apoptotic proteins Bcl-2 and Mcl-1 and to down regulate pro-apoptotic proteins Bim and PUMA (18).

## IL-7 treatment increases T cell function and reverses T cell exhaustion

Immunosuppression with T cell exhaustion is a hallmark in oncology and leads to worsened clinical outcomes (3, 14). IL-7 potently activates lymphocytes by stimulating the JAK-STAT pathway and can prevent or reverse T cell exhaustion (7, 8, 27). Pachynski and colleagues tested the *cellular* vaccine sipuleucel-T with or without IL-7 in patients with metastatic prostate cancer (14). Patients who received sipuleucel-T plus IL-7 had increased T and NK cell functionality and diminished T cell anergy markers compared with those from control patients. Tilsed and colleagues recently reported that IL-7 more than doubled the effect of mRNA-mediated protein production in CD4 and CD8 T cells in an *in vivo* murine model (28). This effect did not occur when either IL-2 or IL-15 was used. Importantly, IL-7 may also restore or amplify the efficacy of checkpoint inhibitors. Work from the Wherry laboratory showed that IL-7 increased lymphocyte cytokine production (polyfunctionality) when used in combination with anti-PD-L1 whereas anti-PD-L1 alone had no effect (27).

Life-threatening infections are a frequent occurrence in oncology patients and tend to exacerbate existing immunosuppression; IL-7 can mitigate immunosuppression (29). T cell IFN-γ production is significantly impaired in patients with sepsis and likely contributes to the failure to eradicate the invading pathogens. An *ex vivo* study of blood from patients with septic shock showed that IL-7 reversed the impaired T cell function and caused a 172 ± 77% increase in the number of IFN-γ producing lymphocytes compared to controls (30). Case reports of patients with life-threatening bacterial and/or fungal sepsis who were treated on a compassionate basis with IL-7 demonstrated that IL-7 caused a greater than 50% increase in IFN-γ production and was accompanied by clearance of the infections (31, 32).

## IL-7 treatment increases stem-like CD4 and CD8 T cells

Stem-like CD4 and CD8 T cells are critical in cancer therapy because they have the capability of self-renewal and can generate large populations of T effector cells upon antigen re-encounter thereby leading to a sustained anti-tumor immune response (3, 15). IL-7 has unique capabilities to potently expand both stem-like CD4 T and stem-like CD8 T cells (3, 8). A phase II trial in 47 patients with advanced urothelial cancer testing atezolizumab (anti-PD-L1) in combination with either IL-7 or placebo showed that patients who received atezolizumab plus IL-7, but not atezolizumab alone, showed a seven-fold and twelve-fold increase in stem-like CD4 and CD8 T cells respectively (15). This increase was associated with a doubling of the complete response rate and durable responses exceeding 2 years in a subset of patients.

## IL-7 treatment does not increase immunosuppressive T regulatory cells

T regulatory cells (T regs) prevent effective anti-tumor responses by multiple mechanisms including secretion of immunosuppressive cytokines, reduction of co-stimulatory signals, and down regulation of antigen-presentation by dendritic cells (5, 12, 18). IL-2 binds preferentially to the high-affinity IL-2 receptor alpha (IL-2Rα, CD25), which stimulates production of T regs and thereby inhibits the tumor-killing effect of adoptive T cell therapies. Foxp3^+^ regulatory T cells are largely unresponsive to IL-7 because they express very low or undetectable levels of the IL-7 receptor alpha chain (CD127) on their surface (33, 34). The downregulation of the expression of the IL-7 receptor alpha chain is directly mediated by Foxp3 which represses IL7R transcription (33, 34).

## IL-7 treatment enhances T cell receptor diversity and epitope spreading

Tumor relapse often results from antigen escape whereby malignant cells downregulate or lose the targeted antigens (3, 8, 9). TCR diversity refers to the breadth of TCR clonotypes that are present. A more diverse TCR repertoire increases the probability that the patients’ T cells will recognize rare or novel tumor “neo-antigens” that occur beyond the original target antigen (9–12). Thus, by increasing TCR diversity, IL-7 increases the likelihood of epitope spreading. Increased TCR diversity is particularly critical where antigen heterogeneity and immune evasion pose significant challenges, as occurs more frequently in solid tumors and during tumor killing in which there is release of novel tumor antigens. IL-7 induces polyclonal T cell expansion of naïve and memory T cells thereby broadening TCR diversity and increasing the likelihood that the immune response extends beyond the original target antigen to include new or secondary tumor antigens (9, 19). In a study of lymphopenic HIV-infected patients, IL-7 caused an approximate 3-6-fold increase in detectable low frequency clonotypes (10, 11). Other investigators noted an approximate 25-50% increase in receptor diversity metrics (12). IL-7 also promotes cross-priming by expanding CD4 T helper cells and boosting CD8 T cell survival and responsiveness (18). By amplifying the attack against existing tumor antigens and by increasing the recognition of novel tumor antigens, IL-7 has the potential to decrease the risk of tumor relapse and enhance the efficacy of the various immune cell therapies, i.e. CAR-T, TIL, and endogenous cell therapies.

## IL-7 treatment enhances trafficking into and throughout tumors

A major limitation of cell therapies in the treatment of solid tumors is poor tumor penetrability (35). This is particularly true in certain tumors, e.g. pancreatic tumors, in which there are dense stromal elements. IL-7 upregulates integrins, for example, LFA-1 and VLA-4, and increases homing chemokine receptors, e.g., CXCR3 and CCR7, which facilitate T cell tumor trafficking (36–38). Improved T cell trafficking translates into greater tumor infiltration and sustained immune pressure on malignant cells. This effect of IL-7 to induce T cell trafficking is demonstrated most clearly by the prompt dramatic *decrease* in circulating lymphocytes (absolute lymphocyte count) that occurs within 4–8 hours after IL-7 administration, which is due to lymphocytes leaving the circulation and trafficking to sites of inflammation or infection (10, 19, 37). The IL-7-induced *decrease* in the absolute lymphocyte count is frequently as great as 40-50% before returning to baseline 36–48 hours later (10, 16, 36). The ability of IL-7 to increase T cell tumor trafficking was recently highlighted by Singh et al. who reported that IL-7 caused a 40-fold increase in the number of CD8 T cells present in a murine glioblastoma model (38). Surprisingly, IL-2 failed to increase CD8 T cell trafficking into the glioblastoma tumor. IL-7 not only increases lymphocyte trafficking to tumors but also to sites of infection (31). IL-7 was used on a compassionate basis in a patient dying of refractory invasive fungal sepsis. Immunohistochemical staining of wound margins at the site of fungal infections showed that the number of CD3 positive lymphocytes present increased by 3–4 fold shortly after beginning IL-7 therapy and was associated with rapid clearance of fungal pathogens from blood and tissue cultures and clinical recovery.

## IL-7 treatment may reduce infectious complications in immune cell therapies

Patients receiving adoptive T cell therapies are often heavily pretreated with lympho-depleting drugs and consequently are at high risk for opportunistic infections. Grade 3–4 infections occur in up to 30% of patients undergoing CAR-T cell therapy, representing a major cause of morbidity and mortality (29). IL-7 enhances host immunity by multiple overlapping mechanisms including: i) restoring CD4 and CD8 T cell counts and functional activity, ii) increasing TCR diversity, iii) reversing T cell exhaustion, iv) preventing apoptosis-induced immune suppression, and v) increasing mucosal-associated invariant T (MAIT) cells which enhance respiratory and intestinal barrier defenses to prevent invading pathogens (36, 39, 40). A recent double-blind, randomized, placebo-controlled trial of IL-7 in 109 critically-ill patients with COVID-19 showed that IL-7-treated patients had 44% fewer hospital-acquired infections versus placebo-treated patients (*p* = 0.014) (17, 18). By reducing infectious complications, IL-7 will improve the safety, outcomes, and quality of life for patients undergoing cell therapies.

## IL-7 treatment may decrease the need for lymphodepleting regimens

Currently, chemotherapy and/or radiation therapy are used to lymphodeplete patients to create “space” for the infused adoptive cell therapies to expand. However, lymphodepletion carries risks of prolonged bone marrow suppression, increases risks of infectious complications, and has a potential long-term toxicity. By enhancing lymphocyte survival and promoting lymphocyte homeostatic proliferation, investigators have postulated that IL-7 may enable adoptively transferred cells to engraft and expand either without the need for preparatory intense lymphodepleting therapies or by enabling a much less toxic lymphodepleting regimen (28, 41, 42). At the present time there are no confirmatory studies in patients, and it is likely that lymphodepletion will continue to be necessary in certain settings. However, it is quite possible that the degree of lymphodepleting agents could be significantly reduced if IL-7 is effective at hastening bone marrow recovery. Studies by Perales et al. support the ability of IL-7 to induce a more rapid immune reconstitution (9). The investigators reported that IL-7 accelerated recovery of *functional* CD4 and CD8 T cells in 12 patients who underwent T-cell-depleted allogenic hematopoietic stem cell transplantation without causing significant graft versus host disease or other serious toxicity.

## IL-7 therapy is well-tolerated and is unlikely to induce cytokine storm

There are well over a dozen clinical trials of IL-7 in more than 620 adult and pediatric patients and, in general, IL-7 has been well tolerated with a favorable safety profile when administered by the intramuscular (IM) or subcutaneous (SQ) route (11–17). IL-7 cannot be administered by the intravenous route because of unique pharmacokinetic and pharmacodynamic properties due to receptor mediated clearance that results in plasma levels of IL-7 that are 50–100 fold greater than levels occurring after IM or SQ administration (43). Injection site reactions, low grade fever, and myalgias are the only common adverse side effects resulting from IM or SQ administration. Transient reversible respiratory distress has been reported in patients who had significant pre-existing underlying respiratory disease (16, 17). Studies in patients with sepsis and COVID-19 have shown that IL-7 did not increase circulating levels of the pro-inflammatory cytokines IL-6 or TNF-α compared to patients who received placebo (17). This finding is likely due to the fact that the IL-7 receptor alpha (IL-7R-α, CD127) is significantly downregulated on activated effector T cells thereby making these cell less likely to be stimulated by IL-7 administration (18). In contrast, IL-2 and IL-15 receptors are continually expressed on effector T cells thereby potentially resulting in the production of the pro-inflammatory cytokines IL-6 and TNF-α and increasing the likelihood of cytokine release syndrome.

Because IL-7 activates adaptive immunity, IL-7 is contraindicated in patients with autoimmune diseases including for example, Crohn’s disease, lupus, myasthenia gravis, rheumatoid arthritis, etc. IL-7 is also contraindicated in patients who have T cell malignancies because IL-7 therapy could cause the malignant T cells to undergo more rapid proliferation. IL-7 is also contraindicated in patients who have undergone solid organ and T cell replete allogenic stem cell transplantation.

## Incorporating IL-7 into the CAR-T genome – a prudent idea?

Recently, several groups have reported that transgenic expression of IL-7, either alone or with co-expression of CCL19, in CAR-T cells has resulted both in enhanced CAR-T cell persistence and in improved anti-tumor response (44–49). Of note, given that IL-7 upregulates numerous lymphocyte chemokines, it is questionable if it is necessary to include co-expression of CCL19 (37, 38). Pang et al. reported preliminary findings in patients with ovarian, pancreatic, and hepatocellular malignancies who were treated with IL-7 and CCL19-secreting CAR-T cell therapies positive for glypican-3 or mesothelin (44). The authors reported that the adverse effects of the modified CAR-T cells were similar to conventional CAR-T cell therapies and that there was significantly enhanced anti-tumor activity. Lei et al. tested anti-CD19 CAR-T cells expressing inducible IL-7 and CCL19 in 39 patients with relapsed or refractory large B-cell lymphoma (46). Grade 3 CRS occurred in 12.8% of patients and the overall response rate at 3 months post-single infusion was 79.5% (complete remission, 56.4%, partial response 23.1%). The authors concluded that the modified CAR-T cells could induce durable responses with a manageable safety profile.

Although IL-7 expressing CAR-T cells (or CAR-T cells overexpressing a constitutively active form of the IL-7 receptor) may ultimately be proven to be safe and effective, the authors believe that administration of IL-7 as a separate drug therapy, i.e., not via IL-7 secreting CAR-T cells, may be preferable. Currently, there is concern that CAR-T cells may undergo malignant transformation and incorporation of IL-7, a potent lymphocyte growth factor, may further increase this risk. Additional concerns include difficulty in treating a potential cytokine release syndrome in the setting of uncontrolled and ongoing production of IL-7. Administration of IL-7 as a separate drug therapy, as opposed to IL-7 secreting CAR-T cells, thus enables investigators to decide on the ideal time and dose of administration of IL-7. Finally, there is increasing evidence that continuous stimulation of the JAK/STAT pathway may lead to T cell exhaustion, further supporting an intermittent approach with exogenous IL-7.

## IL-7 and IL-15 – potential partners in immune cell therapy?

While the authors believe that IL-7 has the greatest potential as an immune cell adjuvant, for the many reasons previously discussed, IL-15, another common γ-chain cytokine, may also be beneficial in this setting. Unlike IL-7 which preferentially expands naïve, central memory, and stem-like CD4 and CD8 T cells, IL-15 drives rapid proliferation of cytotoxic CD8 T cells, NK cells, γδ T cells, and NKT cells (50, 51). A key advantage of IL-15 compared to IL-7 is its efficacy at generating highly cytolytic effector populations with vigorous granzyme B and perforin production. This effect of IL-15 may be particularly advantageous for early tumor debulking in patients with large tumor burdens. IL-7, however, possesses several advantages over IL-15. IL-7 produces a more profound increase in stem-like CD4 and CD8 T cells, effectively expands CD4 T helper cells, a population essential for sustaining long-term anti-tumor immunity, and is superior at broadening the T cell repertoire thereby supporting epitope spreading and protection against antigen escape. Although no head-to- studies have been conducted comparing the effects of IL-7 vs IL-15 to increase tumor trafficking, IL-15 can also strongly enhance tumor trafficking via its effects to increase lymphocyte adhesion molecules and chemokines.

A major advantage of IL-7 is its tolerability and low likelihood of inducing the cytokine release syndrome. The excellent tolerability of IL-7 is due in part to the fact that activated CD4 and CD8 effector T cells, which are the major sources of pro-inflammatory cytokines during the CRS, rapidly downregulate IL-7Rα following TCR stimulation. In contrast, IL-15Rβ remains highly expressed on activated CD8 T cells and NK cells thereby rendering these cells able to be potently stimulated by administration of IL-15 to release pro-inflammatory cytokines and induce the cytokine release syndrome. Thus, while IL-15 is an appealing cytokine for amplifying immediate cytotoxic potential, IL-7 has better tolerability and exerts broader, more durable effects that favor long-term immune persistence and tumor control. The authors believe that as better methods to control the cytokine release syndrome are developed, combination therapy with IL-7 and IL-15 will emerge as a synergistic approach. Moreover, because IL-7 expands CD4 T helper cells and restores their cytokine-producing capacity, IL-7 may enhance the survival, differentiation, and metabolic fitness of IL-15 driven cytotoxic lymphocytes, reducing the risk of effector exhaustion and premature attrition.

## Conclusion and future directions

In conclusion, IL-7 works by multiple independent mechanisms to directly address significant limitations of CAR-T, TIL, and endogenous T cell therapies by expanding and sustaining critical T cell populations, enhancing TCR diversity, promoting epitope spreading, reversing T cell exhaustion, enhancing lymphocyte tumor trafficking, increasing stem like CD4 and CD8 T cells, decreasing the risk of serious infectious, and potentially eliminating the need for toxic lymphodepletion in certain settings. These attributes position IL-7 as an ideal immune adjuvant for the next generation of cellular immunotherapies. The integration of IL-7 into adoptive therapy regimens should be prioritized in prospective clinical trials to maximize the curative potential of cellular immunotherapy. Given that the first rule of medicine is “primum non nocere”, administration of IL-7 as a separate drug therapy rather that employing IL-7 secreting CAR-T cells or using CAR-T expressing a constitutively active IL-7 receptor may be the preferred treatment modality. Given the multiple beneficial immune effects that IL-7 can mediate in both settings of oncology and infectious disease, the continued clinical development of IL-7 combination studies holds great promise.

## Data Availability

The original contributions presented in the study are included in the article/supplementary material. Further inquiries can be directed to the corresponding author.
